# Interface stress transfer model and modulus parameter equivalence method for composite materials embedded with tensile pre-strain shape memory alloy fibers

**DOI:** 10.1371/journal.pone.0302729

**Published:** 2024-05-14

**Authors:** Yizhe Huang, Xueliang Duan, Jun Wang, Zhifu Zhang, Yuanyuan Shi, Bin Huang, Enyong Xu

**Affiliations:** 1 School of Mechanical Engineering, Hubei University of Technology, Wuhan, China; 2 State Key Laboratory of Digital Manufacturing Equipment and Technology, Huazhong University of Science and Technology, Wuhan, China; 3 Dongfeng Liuzhou Motor Co., Ltd., Liuzhou, China; 4 School of Mechanical and Electrical Engineering, Hainan University, Haikou, China; Semnan University, ISLAMIC REPUBLIC OF IRAN

## Abstract

The constitutive model and modulus parameter equivalence of shape memory alloy composites (SMAC) serve as the foundation for the structural dynamic modeling of composite materials, which has a direct impact on the dynamic characteristics and modeling accuracy of SMAC. This article proposes a homogenization method for SMA composites considering interfacial phases, models the interface stress transfer of three-phase cylinders physically, and derives the axial and shear stresses of SMA fiber phase, interfacial phase, and matrix phase mathematically. The homogenization method and stress expression were then used to determine the macroscopic effective modulus of SMAC as well as the stress characteristics of the fiber phase and interface phase of SMA. The findings demonstrate the significance of volume fraction and tensile pre-strain in stress transfer between the fiber phase and interface phase at high temperatures. The maximum axial stress in the fiber phase is 705.05 MPa when the SMA is fully austenitic and the pre-strain increases to 5%. At 10% volume fraction of SMA, the fiber phase’s maximum axial stress can reach 1000 MPa. Ultimately, an experimental verification of the theoretical calculation method’s accuracy for the effective modulus of SMAC lays the groundwork for the dynamic modeling of SMAC structures.

## 1. Introduction

Some traditional materials have struggled to meet the demands of various industries for their specific strength, stiffness, and other properties as science and technology have advanced. The emergence of composite materials has greatly aided in the development of materials by resolving these issues to a greater extent [1–3]. Metal matrix composites have drawn significant attention from various industries, particularly heavy industries, and are increasingly used in many fields such as aerospace, the automobile industry, biomedicine, and electronic packaging. This is due to their high specific strength and stiffness [4–7]. SMA is a brand-new class of smart material that possesses special qualities like superelasticity, temperature phase transition, and pre-strain. SMAC, which are composed of SMA and matrix materials, can fully exploit the phase transition and reinforcement attributes of SMA to increase the strength and kinetic properties of the composites [8–12]. Currently, there are two primary problems with composite plates using shape memory alloys (SMAs): firstly, the modulus of the SMA and the substrate varies significantly. In addition, the nickel-titanium SMA used in this article is usually wrapped in the form of fibers in the matrix material composites [13]. How to consider the substrate material and SMA discontinuities during modeling, as well as the stress and displacement transfer between the material interface at the micro level [14, 15]. Secondly, SMAs have unique pre-strain properties and martensite austenite transformation properties [16–18], which not only help to improve the overall stiffness and dynamic properties of composite plates but also offer a fresh approach to designing and managing their vibration and acoustic radiation properties [19, 20].

The constitutive model and modulus parameter equivalence of composite materials are the foundation for the dynamic modeling of composite plates, which directly affects their modeling accuracy and dynamic characteristics [21, 22]. In the past 30 years, scholars from various countries have constructed different types of intrinsic structure relationships for SMA materials from different perspectives, including fine-scale thermodynamic models, fine-scale mechanical models, and macroscopic image-only models [23–26]. Among them, the macroscopic image-only theoretical models based on thermodynamics and phase transition dynamics are more maturely studied [27–29]. Typical image-only intrinsic models include the Tanaka model, the Liang-Rogers model, and the Brinson model, among which the Tanaka and Liang-Rogers models cannot effectively describe the martensitic selective orientation process of SMAs [30]. Brinson, based on the work of Tanaka and Liang, divided the martensite volume fraction into a stress-induced volume fraction and a temperature-induced volume fraction, effectively overcoming the shortcomings of the Tanaka and Liang-Rogers models [31, 32]. The Brinson model is currently the most widely used intrinsic model in engineering.

For composite plate materials embedded with SMAs, some scholars have studied the intrinsic structure models of SMAC applicable to different cases in recent years. For SMAs in the finite strain range, a thermodynamically based three-dimensional image-only intrinsic model was proposed for predicting the hyperelasticity and shape memory effects of SMAC [33, 34]. The intrinsic structure model of SMAC considering continuous time variation was studied to reveal the time-varying properties of SMAC for the problems of time variation of SMA composite properties and fatigue [35]. A neural network-based intrinsic structure relationship for SMAC was investigated in the presence of SMA response stresses [36]. In addition, a three-dimensional thermodynamic intrinsic structure model applicable to arbitrarily shaped objects was investigated for the variation of composite structure shapes and can be downscaled to achieve an accurate description of structures such as wires, rods, beams, and shells [37].

Numerous studies on fiber-reinforced composites have considered the impact of interfacial phases, and theoretical models consisting of two or three phases have been created for various interfacial bonding conditions. The descriptions of the mechanical properties of the interfacial phase in these three-phase theoretical models are different. Among them, Zhang and He [38] investigated the stress transfer and macroscopic mechanical response of carbon nanotube-reinforced composite interfaces by adopting a triple homogeneous axial cylindrical shell shear hysteresis model. The interfacial phase of the fiber-matrix bond was regarded as an isotropic homogeneous material, and the results showed that an increase in the interfacial phase thickness would significantly reduce the stress transfer efficiency. Yao et al. [39] characterized the non-homogeneous properties of the interfacial phase by varying its elastic modulus along the fiber radius direction, based on earlier research, and considered the fiber-matrix-bonded interfacial phase to be a non-homogeneous material. Based on the theory of interfacial shear stress transmission, they also discussed the influence of the gradient of the elastic modulus of the interfacial phase on microscopic stress transfer. Rodiruez-Ramos et al. [40] used finite element simulations to assess the same influence of various interfacial phase parameters on the composite representative body unit’s overall effective modulus of elasticity.

Nevertheless, there currently aren’t any studies that examine the intrinsic structural interaction of non-uniform SMA reinforcement materials that fully take into account the interfacial phase and SMA tensile strain. Comprehending how to take interface stress transfer, SMA pre-strain, and phase transition characteristics into account is essential to revealing the stress transfer characteristics and managing the constitutive interactions of SMA composite materials. This innovative work necessitates firmly and comprehensive research.

The physical homogenization model for periodic non-uniform composite materials with SMA multiphase or multi-size reinforcements will be proposed in this article based on homogenization concepts. Additionally, a macroscopic equivalent model of non-uniform materials taking into account interface phases will be established, and expressions for axial stress and interphase shear stress of fibers, interface phases, and substrates in SMA composite materials will be derived. Utilize numerical simulation to examine the stress transfer properties and macroscopic mechanical behavior of SMA composite materials. On this basis, experimental research on macroscopic modulus was conducted through the trial manufacture of SMA composite material model samples, establishing the groundwork for the dynamic modeling and sound quality research of SMA composite structures.

## 2. Method

### 2.1 Homogenization of SMAC

**Fig 1:**
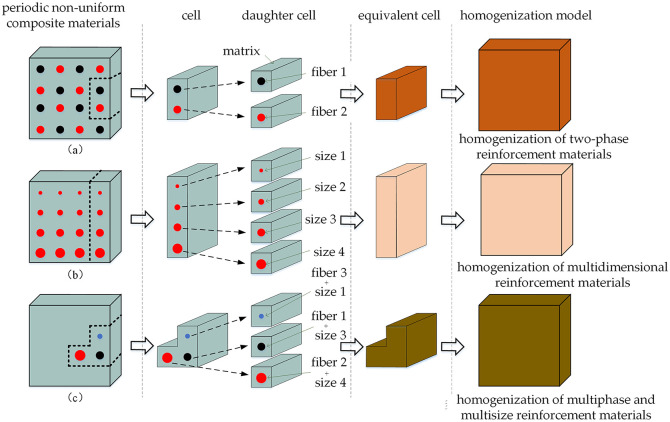
Physical model of homogenization for the multiphase periodic nonuniform composite materials embedded with SMA.

The homogenization method is a processing method that gauges the macroscopic performance of non-uniform materials by analyzing their microscopic attributes and microscopic field. Equivalence can be attained by homogenizing the microscopic structure when the macroscopic characteristic size of non-uniform materials is significantly bigger than the microscopic characteristic size. The physical model of homogenization for the multiphase periodic nonuniform composite materials embedded with of SMA is depicted in Fig 1. Images (a), (b), and (c) portray the two-phase reinforced material structure, the single-phase reinforced material structure with a multi-size gradient distribution, and the multiphase mixed reinforced material structure, respectively.

Assuming that multiphase and multiscale materials are made up of k-type cell representative bodies, the set of cells is made up of k-type cell bodies, the area occupied by different types of cells is *Ω*_1_, *Ω*_2_⋯*Ω*_*k*_, the corresponding volumes of each area are $V_{\Omega_{1}},\;V_{\Omega_{2}}\cdots V_{\Omega_{k}}$, and the cell microscopic stresses and strains are $\sigma_{ij}^{\left(1\right)},\sigma_{ij}^{\left(2\right)}\cdots \sigma_{ij}^{\left(k\right)}$ and $\varepsilon_{ij}^{\left(1\right)},\varepsilon_{ij}^{\left(2\right)}\cdots \varepsilon_{ij}^{\left(k\right)}$, respectively. Considering the impact of the interface phase, the volume average of the density of stress, strain, and strain energy of multiphase and multisize heterogeneous materials may be defined as follows for composites, including multiphase and multisize reinforcing fibers [41].


$${\bar{\sigma }}_{ij}=\frac{1}{\sum_{r=1}^{k}V_{\Omega_{r}}}\overset{k}{\underset{r=1}{\sum }}\left[\int_{\Omega_{rf}}{\left(\sigma_{f}\right)}_{ij}^{\left(r\right)}d\Omega +\int_{\Omega_{ri}}{\left(\sigma_{i}\right)}_{ij}^{\left(r\right)}d\Omega +\int_{\Omega_{rm}}{\left(\sigma_{m}\right)}_{ij}^{\left(r\right)}d\Omega \right]$$ (1)

$${\bar{\varepsilon }}_{ij}=\frac{1}{\sum_{r=1}^{k}V_{\Omega_{r}}}\overset{k}{\underset{r=1}{\sum }}\left[\int_{\Omega_{rf}}{\left(\varepsilon_{f}\right)}_{ij}^{\left(r\right)}d\Omega +\int_{\Omega_{ri}}{\left(\varepsilon_{i}\right)}_{ij}^{\left(r\right)}d\Omega +\int_{\Omega_{rm}}{\left(\varepsilon_{m}\right)}_{ij}^{\left(r\right)}d\Omega \right]$$ (2)

$${\bar{W}}_{ij}=\frac{1}{\sum_{r=1}^{k}V_{\Omega_{r}}}\overset{k}{\underset{r=1}{\sum }}\frac{1}{2}\left[\int_{\Omega_{rf}}{\left(\sigma_{f}\right)}_{ij}^{\left(r\right)}{\left(\varepsilon_{f}\right)}_{ij}^{\left(r\right)}d\Omega +\int_{\Omega_{ri}}{\left(\sigma_{i}\right)}_{ij}^{\left(r\right)}{\left(\varepsilon_{i}\right)}_{ij}^{\left(r\right)}d\Omega +\int_{\Omega_{rm}}{\left(\sigma_{m}\right)}_{ij}^{\left(r\right)}{\left(\varepsilon_{m}\right)}_{ij}^{\left(r\right)}d\Omega \right]$$ (3)

Notation ${\left(\sigma_{f}\right)}_{ij}^{\left(r\right)},{\left(\sigma_{i}\right)}_{ij}^{\left(r\right)}$, and ${\left(\sigma_{m}\right)}_{ij}^{\left(r\right)}$ indicate the stresses in the *r*-th kind of cell’s fibers, interface phases, and matrix materials, respectively;${\left(\varepsilon_{f}\right)}_{ij}^{\left(r\right)},{\left(\varepsilon_{i}\right)}_{ij}^{\left(r\right)}$, and ${\left(\varepsilon_{m}\right)}_{ij}^{\left(r\right)}$ represent the strain of the fibers, interface phase, and matrix material of the r-th kind of cell, respectively; *Ω*_*rf*_, *Ω*_*ri*_, and *Ω*_*rm*_ denote the regions in the fibers, interface phases, and matrix materials of the r-th type of cell, respectively.

The macroscopic effective modulus of non-uniform materials ${\bar{\;E}}_{ijkl}$ can be calculated using Hooke’s law [42]:

$${\bar{\sigma }}_{ij}={\bar{E}}_{ijkl}{\bar{\varepsilon }}_{kl}$$ (4)

Another source of the effective elastic modulus is the strain energy density.


$${\bar{E}}_{ijkl}=\frac{2{\bar{W}}_{ij}}{{\bar{\varepsilon }}_{ij}{\bar{\varepsilon }}_{kl}}$$ (5)

### 2.2 Interface stress transfer model

**Fig 2:**
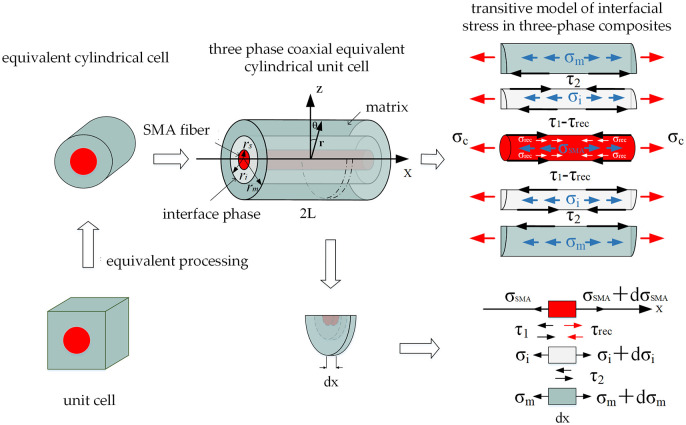
Unit cell physical model of equivalent stress transfer in three-phase coaxial cylindrical model.

The periodic composite material embedded with tensile pre-strain shape memory alloy fibers falls within the category of non-uniform material architectures. Compared to conventional fiber-reinforced materials, it not only has problems with stress transfer between SMA fibers and the matrix material, but also with pre-strain characteristics, phase transition characteristics, and other problems that have an impact on the macroscopic performance of the composite material. To construct a mathematical model for the unit cell of SMA composite materials, taking into account the uniform material and consistent performance of the peripheral parts of the matrix phase, the rectangular cell in section 2 adopts the principle of equal volume, and is treated as a coaxial cylindrical cell similar to that of the SMA fiber reinforced composite material. Interface phase are added to create a three-phase coaxial cylindrical model of unit cell, as shown in Fig 2.

Fig 2 uses a set of cylindrical coordinates (*r*, *θ*, *x*) to describe the equivalent cylindrical cell and assumes the length of the cylindrical model is 2*L* and the radii of SMA fibers, interface phases, and matrix are *r*_*SMA*_, *r*_*i*_, and *r*_*m*_. In addition, SMA fibers in the composite material follow the *x*-direction, and axial tensile stress *σ*_*c*_ is applied to both ends of the cylindrical model.

The fiber and matrix phases of a composite material will both bear axial loads when external loads are applied to both ends if the bonding between the SMA fibers and matrix materials is flawless. The interface phase within the matrix will also bear axial loads and transmit shear stresses. The stress transfer diagram of the matrix materials, interface phases, and SMA fiber in a cylindrical cell is shown in Fig 2. By derivation, the equilibrium equation for SMA fiber, interface phase, and matrix may be determined by factoring in the recovery stress $\sigma_{SMA}^{rec}$ brought on by tensile pre-strain in the SMA fibers.


$$\left(\pi {r_{SMA}}^{2}\right)\sigma_{SMA}+\left[\left(2\pi r_{SMA}\right)dx\right]\left(\tau_{1}-\tau_{rec}\right)=\left(\pi {r_{SMA}}^{2}\right)\left(\sigma_{SMA}+d\sigma_{SMA}\right)$$ (6)

$$\pi \left({r_{i}}^{2}-{r_{SMA}}^{2}\right)\sigma_{i}-\left[\left(2\pi r_{SMA}\right)dx\right]\left(\tau_{1}-\tau_{rec}\right)-\left[\left(2\pi r_{i}\right)dx\right]\tau_{2}=\pi \left({r_{i}}^{2}-{r_{SMA}}^{2}\right)\left(\sigma_{i}+d\sigma_{i}\right)$$ (7)

$$\pi \left({r_{m}}^{2}-{r_{i}}^{2}\right)\sigma_{m}+\left[\left(2\pi r_{i}\right)dx\right]\tau_{2}=\pi \left({r_{m}}^{2}-{r_{i}}^{2}\right)\left(\sigma_{m}+d\sigma_{m}\right)$$ (8)

In Eqs (6) and (7), $\tau_{rec}=\left(r_{SMA}/2l\right)\sigma_{SMA}^{rec}$ is the equivalent shear stress between the fiber filament and the interface under the pre-tensile stress of SMA.

If the SMA fiber is of equal length to the matrix, the boundary conditions of the fiber, interface phase, and matrix are:

$${\left.\sigma_{SMA}\right|}_{x=\pm l}=\sigma_{c},{\left.\sigma_{i}\right|}_{x=\pm l}=\sigma_{c},{\left.\sigma_{m}\right|}_{x=\pm l}=\sigma_{c}$$ (9)

The corresponding cylindrical unit cell implanted with pre-strained SMA fibers will cause recovery stress in the composite due to the restrictions of the surrounding matrix materials. Brinson’s constitutive model can be used to compute the recovery stress of SMA fibers under pre-strain and temperature conditions [25].


$$\sigma_{SMA}^{rec}=\left[E_{A}+\xi \left(E_{M}-E_{A}\right)\right]\left(\varepsilon_{SMA}^{pre}-\varepsilon_{L}\xi_{s}\right)\text{+}\Theta \left(T-T_{0}\right)$$ (10)

In the equation $\varepsilon_{SMA}^{pre}$ is the maximum pre-tensile strain, *ε*_*L*_ is the maximum residual strain, *ξ*_*s*_ is the volume fraction of stress-induced martensite, and *Θ* is the thermal elastic modulus of SMA fiber filament. Total volume fraction of martensite *ξ* = *ξ*_*s*_ + *ξ*_*T*_, including stress-induced volume fraction *ξ*_*s*_ and temperature-induced volume fraction *ξ*_*T*_. Elastic modulus *E*_*SMA*_ = *ξ*(*E*_*M*_ − *E*_*A*_)+*E*_*A*_, E_M_ is the elastic modulus of martensite, and *E*_*A*_ is the elastic modulus of austenite.

The stress-strain relationship of the three-phase coaxial cylindrical cell can be established as follows after taking into consideration the pre-strain recovery stress of SMA, the thermal expansion impact of the interface phase, and the matrix:

$$\left\{\begin{array}{c} \varepsilon_{SMA}^{x}\left(r,x\right)\\ \varepsilon_{SMA}^{r}\left(r,x\right)\\ \varepsilon_{SMA}^{\theta }\left(r,x\right) \end{array}\right\}=\frac{1}{E_{SMA}}\left\{\left\{\begin{array}{c} \sigma_{SMA}^{x}\left(x\right)\\ \sigma_{SMA}^{r}\left(x\right)\\ \sigma_{SMA}^{\theta }\left(x\right) \end{array}\right\}-\upsilon_{SMA}\left\{\begin{array}{c} \sigma_{SMA}^{r}\left(x\right)+\sigma_{SMA}^{\theta }\left(x\right)\\ \sigma_{SMA}^{\theta }\left(x\right)+\sigma_{SMA}^{z}\left(x\right)\\ \sigma_{SMA}^{x}\left(x\right)+\sigma_{SMA}^{r}\left(x\right) \end{array}\right\}\right\}+\left\{\begin{array}{c} -1\\ \upsilon_{SMA}\\ \upsilon_{SMA} \end{array}\right\}\left\{\frac{\sigma_{SMA}^{rec}}{E_{SMA}}\right\}$$ (11)

$$\left\{\begin{array}{c} \varepsilon_{i}^{x}\left(r,x\right)\\ \varepsilon_{i}^{r}\left(r,x\right)\\ \varepsilon_{i}^{\theta }\left(r,x\right) \end{array}\right\}=\frac{1}{E_{i}}\left\{\left\{\begin{array}{c} \sigma_{i}^{x}\left(x\right)\\ \sigma_{i}^{r}\left(x\right)\\ \sigma_{i}^{\theta }\left(x\right) \end{array}\right\}-\upsilon_{i}\left\{\begin{array}{c} \sigma_{i}^{r}\left(x\right)+\sigma_{i}^{\theta }\left(x\right)\\ \sigma_{i}^{\theta }\left(x\right)+\sigma_{i}^{x}\left(x\right)\\ \sigma_{i}^{x}\left(x\right)+\sigma_{i}^{r}\left(x\right) \end{array}\right\}\right\}+\beta_{i}\left(T-T_{0}\right)$$ (12)

$$\left\{\begin{array}{c} \varepsilon_{m}^{x}\left(r,x\right)\\ \varepsilon_{m}^{x}\left(r,x\right)\\ \varepsilon_{m}^{\theta }\left(r,x\right) \end{array}\right\}=\frac{1}{E_{m}}\left\{\left\{\begin{array}{c} \sigma_{m}^{x}\left(x\right)\\ \sigma_{m}^{r}\left(x\right)\\ \sigma_{m}^{\theta }\left(x\right) \end{array}\right\}-\upsilon_{m}\left\{\begin{array}{c} \sigma_{m}^{r}\left(x\right)+\sigma_{m}^{\theta }\left(x\right)\\ \sigma_{m}^{\theta }\left(x\right)+\sigma_{m}^{z}\left(x\right)\\ \sigma_{m}^{x}\left(x\right)+\sigma_{m}^{r}\left(x\right) \end{array}\right\}\right\}+\beta_{m}\left(T-T_{0}\right)$$ (13)

The subscripts "*m*", "*i*", and "*SMA*" in the formula stand for the matrix phase, interface phase, and SMA fiber phase, respectively. *T*_0_ is the model’s starting temperature, and *β*_*i*_, *β*_*m*_ are the coefficients of thermal expansion of the interface phase and matrix phase. The terms *υ*_*m*_, *υ*_*i*_, and *υ*_*SMA*_ refer to the Poisson’s ratio of the matrix phase, interface phase, and SMA fiber phase, respectively.

The elastic modulus and Poisson’s ratio of the interface phase cannot be measured experimentally because its thickness is less than that of the fiber phase and matrix. Anifantis [43] makes the assumption that the elastic modulus and Poisson’s ratio vary exponentially over the radial distance in order to calculate the characteristics of these two material parameters.

$$E_{i}\left(r\right)=E_{m}\left\{1+\left[p\frac{E_{A}+\xi \left(E_{M}-E_{A}\right)}{E_{m}}-1\right]\frac{1-\left(r/r_{i}\right)e^{1-\left(r/r_{i}\right)}}{1-\left(r_{SMA}/r_{i}\right)e^{1-\left(r_{SMA}/r_{i}\right)}}\right\}$$ (14)

$$v_{i}\left(r\right)=v_{m}\left\{1+\left[\left(\frac{1-v_{SMA}/v_{m}}{\left(\left(E_{A}+\xi \left(E_{M}-E_{A}\right)/E_{m}\right)-1\right)v_{SMA}/v_{m}}\frac{1-p}{p}+1\right)\frac{v_{SMA}}{{\text{E}}_{m}}-1\right]\frac{1-\left(r/r_{i}\right)e^{1-\left(r/r_{i}\right)}}{1-\left(r_{SMA}/r_{i}\right)e^{1-\left(r_{SMA}/r_{i}\right)}}\right\}$$ (15)

which

$$p={\text{E}}_{i}\left(r\right)/\left[E_{A}+\xi \left(E_{M}-E_{A}\right)\right]$$ (16)

According to the equilibrium equations of Eqs (6), (7) and (8), the equilibrium relationship between axial stress and interfacial shear stress in each phase of the three-phase coaxial cylindrical cell model can be obtained as follows:

$$\left\{\begin{array}{c} \frac{d\sigma_{SMA}}{dx}=\frac{2}{r_{SMA}}\left(\tau_{1}-\tau_{rec}\right)\\ \frac{d\sigma_{i}}{dx}=\frac{2}{r_{i}^{2}-r_{SMA}^{2}}\left[r_{SMA}\left(\tau_{1}-\tau_{rec}\right)+r_{i}\tau_{2}\right]\\ \frac{d\sigma_{m}}{dx}=\frac{2r_{i}}{r_{m}^{2}-r_{i}^{2}}\tau_{2} \end{array}\right.$$ (17)

Among them, *τ*_1_ represents the shear stress at the interface between the SMA fiber phase and the interface phase caused by external forces, and *τ*_2_ represents the interfacial shear stress between the interface phase and the matrix phase.

Based on Eq (17) and considering the continuity conditions of the interfaces between various phases of materials, the stress differential equations of fibers and interface phases in the three-phase coaxial model of SMA composite materials can be obtained through theoretical analysis and derivation.


$$\left\{\begin{array}{l} d^{2}\frac{\sigma_{SMA}}{\sigma_{c}}/dx^{2}=\left(M^{\prime }-M_{11}\frac{\varepsilon_{SMA}^{pre}-\varepsilon_{L}\xi_{s}}{\sigma_{c}}\right)+\left(M_{12}-\frac{r_{SMA}^{2}}{r_{m}^{2}}M^{\prime }\right)\frac{\sigma_{SMA}}{\sigma_{c}}+\left(M_{13}-\frac{r_{i}^{2}-r_{SMA}^{2}}{r_{m}^{2}}M^{\prime }\right)\frac{\sigma_{i}}{\sigma_{c}}\\ d^{2}\frac{\sigma_{i}}{\sigma_{c}}/dx^{2}=\left(M^{\prime \prime }-M_{21}\frac{\varepsilon_{SMA}^{pre}-\varepsilon_{L}\xi_{s}}{\sigma_{c}}\right)+\left(M_{22}-\frac{r_{SMA}^{2}}{r_{m}^{2}}M^{\prime \prime }\right)\frac{\sigma_{SMA}}{\sigma_{c}}+\left(M_{23}-\frac{r_{i}^{2}-r_{SMA}^{2}}{r_{m}^{2}}M^{\prime \prime }\right)\frac{\sigma_{i}}{\sigma_{c}} \end{array}\right.$$ (18)

The coefficients in the equation *M*_*ij*_ (*ij* = 11, 12, 13, 21, 22, 23), *M*′ and *M*″ are as follows:

$$M_{11}=C_{1}\left[\frac{A_{2}B_{1}}{A_{4}}\frac{E_{m}}{1+v_{m}}-\left(B_{1}-1\right)\frac{E_{i}}{1+v_{i}}\right]$$ (19)

$$M_{12}=C_{1}\left[\frac{A_{2}B_{1}}{A_{4}}\frac{E_{m}}{\left(1+v_{m}\right)E_{SMA}}-\left(B_{1}-1\right)\frac{E_{i}}{\left(1+v_{i}\right)E_{SMA}}\right]$$ (20)

$$M_{13}=C_{1}\left[\frac{A_{2}B_{2}}{A_{4}}\frac{E_{m}}{\left(1+v_{m}\right)E_{i}}-\frac{B_{2}}{1+v_{i}}\right]$$ (21)

$$M^{\prime }=C_{1}C_{2}\left[\frac{A_{2}\left(B_{3}-1\right)}{A_{4}}\frac{1}{1+v_{m}}-\frac{B_{3}}{1+v_{i}}\frac{E_{i}}{E_{m}}\right]$$ (22)

$$M_{21}=C_{3}\left[\frac{B_{1}}{A_{4}}\left(1-\frac{A_{2}}{A_{1}}\right)\frac{1}{1+v_{m}}E_{m}+\frac{B_{1}-1}{A_{1}}\frac{E_{i}}{1+v_{i}}\right]$$ (23)

$$M_{22}=C_{3}\left[\frac{B_{1}}{A_{4}}\left(1-\frac{A_{2}}{A_{1}}\right)\frac{E_{m}}{\left(1+v_{m}\right)E_{SMA}}+\frac{B_{1}-1}{A_{1}}\frac{E_{i}}{\left(1+v_{i}\right)E_{SMA}}\right]$$ (24)

$$M_{23}=C_{3}\left[\frac{B_{2}}{A_{4}}\left(1-\frac{A_{2}}{A_{1}}\right)\frac{E_{m}}{\left(1+v_{m}\right)E_{i}}+\frac{B_{2}}{A_{1}}\frac{1}{1+v_{i}}\right]$$ (25)

$$M^{\prime \prime }=C_{2}C_{3}\left[\frac{B_{3}-1}{A_{4}}\left(1-\frac{A_{2}}{A_{1}}\right)\frac{1}{1+v_{m}}+\frac{B_{3}}{A_{1}}\frac{E_{i}}{\left(1+v_{i}\right)E_{m}}\right]$$ (26)

Among them, the expressions for *A*_*p*_(*p* = 1, 2,…, 6), *B*_*q*_(*q* = 1, 2,…, 6), and *C*_*t*_(*t* = 1, 2, 3, 4) are detailed in equations A-1 to A-4 in S1 Appendix. The general solution of the control equation for the three-phase coaxial model can be expressed as:

$$\left\{\begin{array}{c} \frac{\sigma_{SMA}}{\sigma_{c}}=K_{1}+H_{1}\frac{\text{cosh}\left(T_{1}x\right)}{\text{cosh}\left(T_{1}L\right)}+H_{2}\frac{\text{cosh}\left(T_{2}x\right)}{\text{cosh}\left(T_{2}L\right)}\\ \frac{\sigma_{i}}{\sigma_{c}}=K_{2}+J_{1}\frac{\text{cosh}\left(T_{1}x\right)}{\text{cosh}\left(T_{1}L\right)}+J_{2}\frac{\text{cosh}\left(T_{2}x\right)}{\text{cosh}\left(T_{2}L\right)} \end{array}\right.$$ (27)

From this, it can be concluded that the axial stress and interfacial shear stress expressions of fibers, interface phases, and matrix in pre-strained SMA fiber-reinforced composites are:

$$\sigma_{SMA}=\left[K_{1}+H_{1}\frac{cosh\left(T_{1}x\right)}{cosh\left(T_{1}L\right)}+H_{2}\frac{cosh\left(T_{2}x\right)}{cosh\left(T_{2}L\right)}\right]\sigma_{c}$$ (28)

$$\sigma_{i}=\left[K_{2}+J_{1}\frac{cosh\left(T_{1}x\right)}{cosh\left(T_{1}L\right)}+J_{2}\frac{cosh\left(T_{2}x\right)}{cosh\left(T_{2}L\right)}\right]\sigma_{c}$$ (29)

$$\sigma_{m}=\left\{C_{2}-C_{4}\left[K_{1}+H_{1}\frac{cosh\left(T_{1}x\right)}{cosh\left(T_{1}L\right)}+H_{2}\frac{cosh\left(T_{2}x\right)}{cosh\left(T_{2}L\right)}+\left({\left(r_{i}/r_{SMA}\right)}^{2}-1\right)\left(K_{2}+J_{1}\frac{cosh\left(T_{1}x\right)}{cosh\left(T_{1}L\right)}+J_{2}\frac{cosh\left(T_{2}x\right)}{cosh\left(T_{2}L\right)}\right)\right]\right\}\sigma_{c}$$ (30)

$$\tau_{1}=\tau_{rec}-\frac{r_{SMA}}{2}\left\{H_{1}T_{1}\;tanh\left(T_{1}x\right)+H_{2}T_{2}\;tanh\left(T_{2}x\right)\right\}\sigma_{c}$$ (31)

$$\begin{array}{c} \tau_{2}=-\frac{\sigma_{c}r_{SMA}}{2\left(r_{i}/r_{SMA}\right)}\left\{\left({\left(r_{i}/r_{SMA}\right)}^{2}-1\right)\right.\left[J_{1}T_{1}\;tanh\left(T_{1}x\right)+J_{2}T_{2}\;tanh\left(T_{2}x\right)\right]\\ \left.+H_{1}T_{1}\;tanh\left(T_{1}x\right)+H_{2}T_{2}\;tanh\left(T_{2}x\right)\right\} \end{array}$$ (32)

The coefficients in the equations *K*_1_, *K*_2_, *H*_1_, *H*_2_, *T*_1_, *T*_2_, *J*_1_, and *J*_2_ please refer to equations A-5 to A-15 in S1 Appendix. Eqs (28) to (32) can be used to analyze the axial stress and shear stress of a single SMA composite cell in the composite under the external load of the SMA fiber, interface phase, and matrix, reveal its stress-strain and stress transitive relations, and lay a foundation for the research of the macroproperties of the composite.

The macroscopic effective modulus of SMA composite materials depends on the fiber, matrix, and interfacial phase stress-strain relationship as well as the interfacial phase parameter properties in Eqs (14) and (15). Based on the homogenization equivalent method in this chapter and the stress expressions from Eqs (28) to (32), the macroscopic effective elastic modulus of SMAC can be obtained as follows:

$$E_{SMAC}=\left\{1+\left[Q_{1}\left(\frac{E_{m}}{E_{A}+\xi \left(E_{M}-E_{A}\right)}+1\right)+Q_{2}\right]V_{SMA}\right\}E_{m}+V_{SMA}\left[E_{A}+\xi \left(E_{M}-E_{A}\right)\right]$$ (33)

which

$$Q_{1}=\frac{2\sqrt{3}}{\pi }\left[K_{1}+J_{1}R_{1}\frac{\text{tanh}\left(T_{1}L\right)}{T_{1}L}+J_{2}R_{2}\frac{\text{tanh}\left(T_{2}L\right)}{T_{2}L}\right]$$ (34)

$$Q_{2}=\frac{4\sqrt{3}}{\pi }\left[{\left(r_{i}/r_{SMA}\right)}^{2}-1\right]\left[K_{2}+J_{1}\frac{\text{tanh}\left(T_{1}L\right)}{T_{1}L}+J_{2}\frac{\text{tanh}\left(T_{2}L\right)}{T_{2}L}\right]$$ (35)

The equivalent elastic modulus of SMA non-uniform composite materials can be obtained through Eq (33).

## 3. Theoretical analysis results and discussion

### 3.1 Parsing method validation

**Fig 3:**
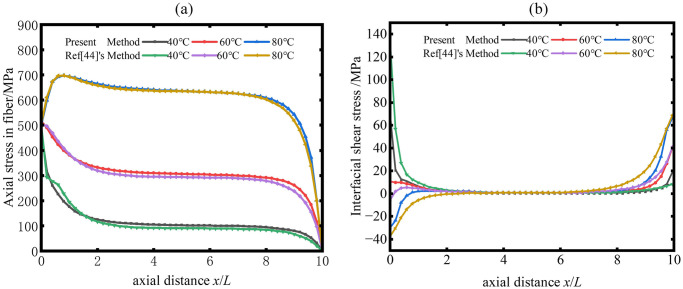
Comparison between present analytical methods and Wang’s simulation results (a) Distribution of axial stresses in fibers; (b) Distribution of shear stress at the interface.

To verify the theoretical formula accuracy of this study, numerical example is described and discussed in predicting the distribution of axial stress and interfacial shear stress without considering tensile pre-strain shape memory alloy fibers. Example for verifying comparison is axial stress and interfacial shear stress comparison of the SMA composite materials subjected to different temperatures (T = 40°C, 60°C, 80°C) are presented in Fig 3, which was taken from Wang et al [44].

Wang et al. used the Fortran language to write a subroutine that can be called by Msc. Marc, which is a large-scale commercial finite element software program. They then used the finite element simulation method to analyze the internal stress distribution of the composite single-fiber model embedded with SMA fiber at various temperatures. As can be observed from Fig 3, the theoretical analytical method of this paper is verified for correctness and accuracy by a high degree of overlap between the results obtained from this method and the curves of the simulation results in reference [44], provided that the same parameters are used.

### 3.2 Materials parameters

Assuming the volume fraction of SMA in the unit cell of SMA composite materials *V*_*SMA*_ = 20%, then $r_{SMA}^{2}/r_{m}^{2}=0.2$. SMA wire radius *r*_*SMA*_ = 0.252*mm*, according to equivalent treatment $\pi r_{m}^{2}=a_{m}^{2}$, the corresponding equivalent unit cell radius of the cylinder is *r*_*m*_ = 0.564 *mm*, and the interface phase radius is set to *r*_*i*_ = 0.3 *mm*. Half of the length of a single cell unit is *L* = 200 *mm*. The material properties of the SMA fiber phase and matrix phase are shown in Fig 3 and Table 1, where the matrix material in the matrix phase is composed of graphite and epoxy resin composites [45].

**Table 1:** Material properties of SMA, graphite, and epoxy resin [46].

SMA		graphite		epoxy resin	
*E*_*SMA*_(*GPa*)	See Fig 4	*E*_*g*_(*GPa*)	275.6	*E*_*e*_(*GPa*)	3.43
$\sigma_{SMA}^{rec}\left(MPa\right)$	See Fig 4	*G*_*g*_(*GPa*)	114.8	*G*_*e*_(*GPa*)	1.27
*v*_*SMA*_	0.33	*v*_*g*_	0.2	*v*_*e*_	0.35
*α*_*SMA*_(1°*C*)	10.26×10^−6^	*α*_*g*_(1°*C*)	24.40×10^−6^	*α*_*e*_(1°*C*)	64.80×10^−6^

Using realistic temperature conditions of 25°C for ambient temperature and 100°C for high temperature, numerical calculations were performed. The corresponding elastic moduli of SMA fibers at room temperature and high temperature are, respectively, *E*_*SMA*_(25°*C*) = 28.755 *GPa* and *E*_*SMA*_(100°*C*) = 81.800 *GPa*. Set the elastic modulus of the corresponding interface phase at room temperature and high temperature as, respectively, *E*_*i*_(25°*C*) = 20 *GPa* and *E*_*i*_(100°*C*) = 70 *GPa*. The elastic modulus of the matrix phase is *E*_*m*_ = 8.873 *Gpa*, and the corresponding Poisson’s ratios for the three phases are *v*_*SMA*_ = 0.33, *v*_*i*_ = 0.34, and *v*_*m*_ = 0.347. At room and high temperatures, add 1%, 3%, and 5% tensile pre-strain to the SMA fiber phase and apply axial stress of 200 *MPa* at the end of a single cell. For simplicity of expression, in the subsequent illustrations of this article, Unified Simplified *V*_*S*_, ε_*p*_, and *σ*_*s*_ represent the volume fraction, pre-strain, and stress symbols.

### 3.3 Distribution of axial stress and interfacial shear stress

Based on the theoretical model in the previous section, it is possible to calculate the axial stress, shear stress at the upper and lower boundaries of the SMA fiber, the interface phase, and the matrix, as well as the difference in shear stress at the interface phase, as shown in Fig 5.

**Fig 4:**
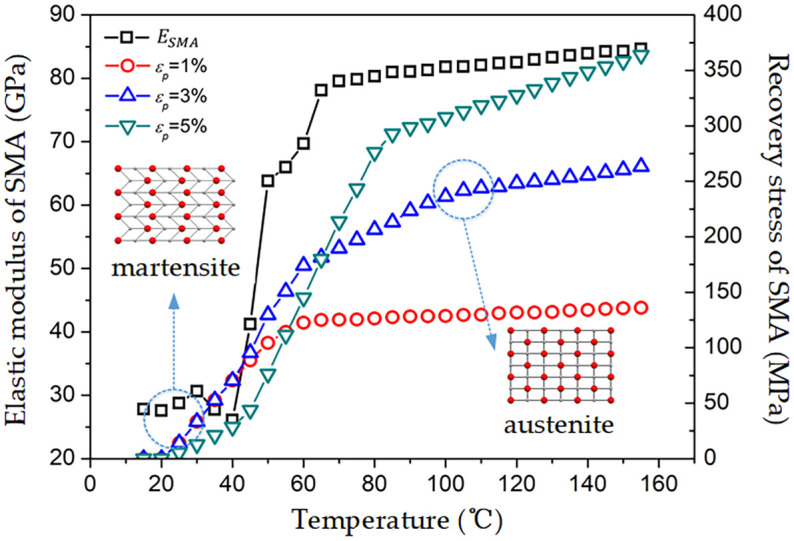
Changes in SMA elastic modulus and recovery stress with temperature.

**Fig 5:**
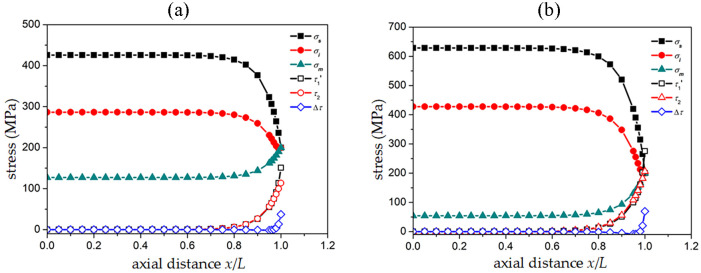
Stress distribution of fiber phase, interface phase, and matrix phase in SMA composite materials: (a) Temperature 25°C; (b) Temperature 100°C.

Defined in Fig 4, $\tau_{1}^{\prime }=\tau_{1}-\tau_{rec},\;\tau_{1}^{\prime }$ refers to the shear stress at the interface between the SMA fiber phase and the interface. The terms *σ*_*s*_, *σ*_*i*_, and *σ*_*m*_ refer to the axial stress of the fiber phase, interface phase, and matrix phase, respectively, while *τ*_2_ refers to the shear stress between the interface phase and the matrix phase. The maximum value of the axial stresses in the cross section in the SMA fiber phase is in the middle of the specimen (x = 0) in Fig 5, which illustrates the axial stresses of each phase of the L/2~L portion. This is because the axial stresses of each phase are symmetric about the middle of the specimen. As the axial distance increases, the axial stress gradually decreases within the range of 0~0.7 at the axial distance *x*/*L*. After the axial distance *x*/*L* exceeds 0.7, the axial stress rapidly decreases to an external stress. The variation pattern of axial stress in the interface phase is similar to that in the SMA fiber phase, but the stress value of the interface phase is smaller than that of the SMA fiber phase. When the axial distance *x*/*L* is greater than 0.7, the matrix phase quickly transitions to external stress. The shear stress at the upper and lower boundaries of the interface phase and the difference in shear stress at the interface ($\Delta \tau =\tau_{1}^{\prime }-\tau_{2}$) as the axial distance changes, the shear stress and shear stress difference increase rapidly near the end region and reach their maximum value at the end. The reason for this is known from Hooke’s law, which states that the larger the modulus of the phase in a cell under a certain strain, the greater the corresponding stress, and the stress transfer at the middle position of the specimen meets Hooke’s law. Near the end face of a cell, due to the same external stress, the axial stress and shear stress of the three phases work together to achieve the same axial stress on the end face. In addition, compared to a room-temperature environment of 25°*C*, a high-temperature environment of 100°*C* results in higher axial and shear stresses corresponding to the fiber phase and interface. This is mainly due to the temperature rise, the SMA fiber undergoes the transformation from the martensite phase to the austenite phase, and the corresponding elastic modulus of the fiber increases from 28.755 *GPa* to 81.800 *GPa*. The increase in the overall modulus of the unit cell increases both the axial stress and the shear stress.

### 3.4 Stress transmission in SMAC as an effect of SMA tensile strain

**Fig 6:**
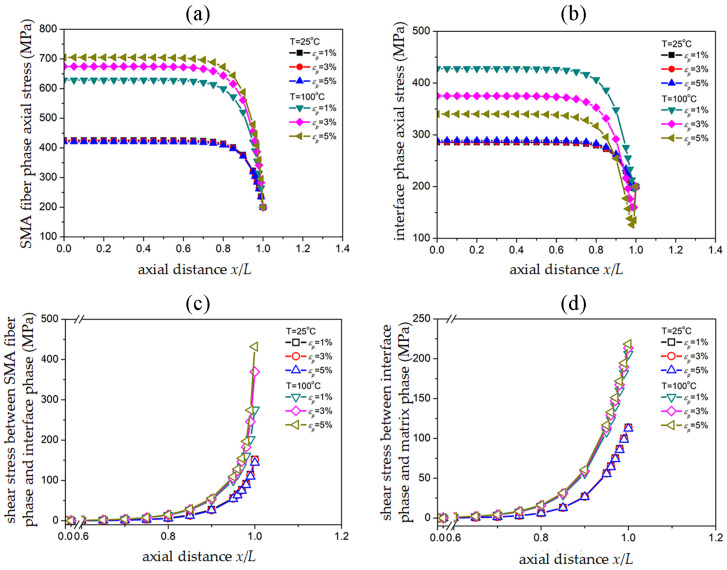
Effect of SMA pre-strain on SMAC stress distribution: (a) Axial stress of SMA fiber phase; (b) Axial stress of interface phase; (c) Shear stress between SMA fiber phase/interface phase; (d) Shear stress between interface phase/matrix phase.

Fig 6(a) and 6(b) show that the volume fraction of SMA fibers is *V*_*SMA*_ = 20% in two temperature environments of 25°C and 100°C, and the SMAC end is subjected to uniform tensile external stress. The axial stress distribution in the SMA fiber phase and interface phase was investigated for three different SMA fibers, subject to a uniform tensile external stress of *σ*_*c*_ = 200 *MPa* and pre-strains of 1%, 3%, and 5%. Fig 6(c) and 6(d) show the shear stress distribution at the upper and lower edges of the interface phase.

Based on Fig 4 and a comparison with the stress distribution curves shown in Fig 6, it was observed that the recovery stress generated under different SMA pre-strain conditions at 25°*C* was minimal and showed little variation. With increasing levels of pre-strain in the SMA at 100°*C*, a greater recovery stress was generated, resulting in decreased overall cell stiffness and increased deformation under unchanged SMA modulus conditions. Moreover, the axial stress in the SMA fiber phase also increased with increasing pre-strain. Therefore, the effect of pre-strain on the maximum axial stress of the SMA fiber phase is significant at high temperatures.

Based on the stress distribution analysis presented in Fig 6, it was observed that the maximum axial stresses of the SMA fiber phase corresponding to pre-strains of 1%, 3%, and 5% at 100°*C* were 628.57 *MPa*, 674.58 *MPa*, and 705.05 *MPa*, respectively, whereas the corresponding maximum axial stresses of the interface phase were measured as 427.76 *MPa*, 375.04 *MPa*, and 340.11 *MPa*, respectively. The differences in stress transfer between the two phases were found to be 200.81 *MPa*, 299.54 *MPa*, and 364.94 *MPa*, respectively. Additionally, the shear stress differences between the upper and lower boundaries of the corresponding interface phases were determined to be 69.49 *MPa*, 156.10 *MPa*, and 213.48 *MPa*, respectively. The stress distribution analysis indicates that pre-strain at high temperatures plays a key role in regulating stress transfer between the fiber and interface phases. This is mainly due to the increase in pre-strain leading to an increase in the maximum axial stress of the fiber phase, while the difference in shear stress between the upper and lower boundaries of the interface phase (shear stress transmission) determines the maximum axial stress of the interface phase. The increase in pre-strain increases the shear stress between the SMA fiber phase and the interface phase, improving the efficiency of stress transfer. It is worth noting that in Fig 6(b), the minimum axial stress of the interface phase at 100°*C* appears in the region of *x*/*L* = 0.9~1.0. The axial location of *x*/*L* = 0.98 corresponds to the minimum axial stress of 126.25 *MPa* when the pre-strain is 5%. As the pre-strain decreases, the minimum axial stress increases, and the corresponding axial position will gradually approach the end.

### 3.5 Stress transmission in SMAC as an effect of SMA volume fraction

**Fig 7:**
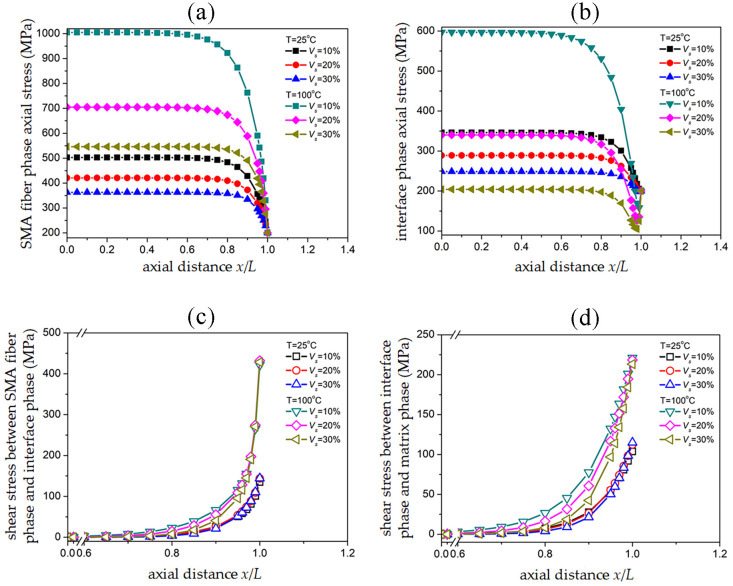
Effect of SMA volume fraction on stress distribution of SMAC: (a) Axial stress of SMA fiber phase; (b) Axial stress of interface phase; (c) Shear stress between SMA fiber phase/interface phase; (d) Shear stress between interface phase/matrix phase.

Fig 7(a)–7(d) illustrate the pre-strain of SMA fibers at two temperatures, 25°*C* and 100°*C* with *ε*_*p*_ = 5%, and the SMAC end being subjected to uniform tensile external stress of *σ*_*c*_ = 200 *MPa*. The distribution of axial stress, interfacial axial stress, and shear stress at the upper and lower edges of three different SMA fiber volume fractions of 10%, 20%, and 30% corresponds to the SMA fiber phase. By altering the diameter of the fibers under the conditions of constant fiber density and single-cell composite material volume, the volume percentage of SMA fibers can be adjusted.

By observing the curves in Fig 7, it can be observed that as the volume fraction of SMA fibers increases, the maximum axial stress of the fiber phase and interface phase gradually decreases. This is mainly because of the higher modulus of SMA compared to the matrix, and the increase in the volume fraction of SMA fibers in a single cell leads to an increase in the overall stiffness of the cell. Under the same external stress, the strain of the cell decreases. At this point, the modulus in the SMA fiber phase remains constant at a constant temperature, resulting in a decrease in the maximum axial stress on the cross-section of the fiber phase. Similarly, the modulus setting of the interface phase is also greater than that of the matrix phase, and the axial stress on the cross-section in the interface phase is also reduced.

Compared with the temperature environment at 25°*C*, the maximum axial stress on the cross-section of the fiber phase significantly increases at 100°*C*, and the volume fraction of SMA fibers with the same increment has a more significant impact on the maximum axial stress. On the one hand, it is due to the phase transformation characteristics caused by temperature rise, which transforms into an austenite phase at high temperatures. The increase in modulus promotes the maximum axial stress to increase. On the other hand, the volume percentage of SMA fibers has a positive correlation with the recovery stress caused by the pre-strain of SMA fibers. As the volume fraction increases, the recovery stress also increases, offsetting the original axial stress and further reducing the maximum axial stress, thus showing a significant difference in the maximum axial stress under different SMA volume fractions.

For the maximum axial stress on the cross-section of the interface phase, at a temperature of 100°*C* and a volume fraction of 10% SMA, the maximum axial stress is significantly greater than the corresponding maximum axial stress at different volume fractions of 20%, 30%, and 25°*C*. Based on Fig 7(a)–7(d), the increase in modulus of SMA fibers due to phase transformation at high temperatures has an increasing effect on the axial stress of the fiber phase and the shear stress of the fiber phase/interface phase. At the same time, the change in the volume fraction of SMA fibers will affect the recovery stress generated by SMA pre-strain. Therefore, the axial stress in the interface phase and the shear stress above and below the interface phase will be redistributed.

The changes in axial stress within a single cell are not only borne by the SMA fiber phase but also by the interface phase and matrix phase. It is clear from Fig 7(c) and 7(d) that at high temperatures, the interface phase can transmit more external stress and that recovery stress has a different effect on localized shear stress.

### 3.6 Stress transmission in SMAC as an effect of graphite volume fraction

**Fig 8:**
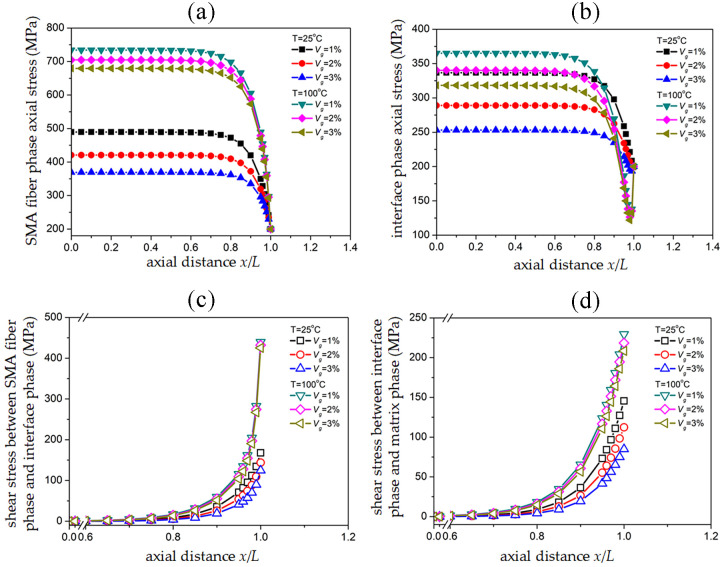
Effect of graphite volume fraction on stress distribution of SMAC: (a) Axial stress of SMA fiber phase; (b) Axial stress of interface phase; (c) Shear stress between SMA fiber phase/interface phase; (d) Shear stress between interface phase/matrix phase.

Fig 8(a)–8(d) show the pre-strain of SMA fibers is 5% and volume fraction *V*_*s*_ = 20% in two temperature environments of 25°*C* and 100°*C*, and the SMAC end is subjected to uniform tensile external stress *σ*_*c*_ = 200 *MPa*, with different graphite volume fractions *V*_*g*_ = 1%, *V*_*g*_ = 2%, and *V*_*g*_ = 3% corresponding to the distribution of axial stress in SMA fiber phase, interface phase, and shear stress at the upper and lower edges of the interface phase.

Matrix elastic modulus after the composite of three graphite volume fractions *E*_*m*_ is 6.152 *GPa*, 8.873 *GPa*, and 11.595 *GPa*, respectively. Compared to the elastic modulus of 81.800 *GPa* for the SMA fiber phase at high temperature and 70 *GPa* for the interface phase, the modulus weight of 28.755 *GPa* for the SMA fiber phase and 20 *GPa* for the interface phase at room temperature is greater in the unit cell. The change in graphite volume fraction has a more significant impact on the overall stiffness of the unit cell. Therefore, at room temperature, the change in graphite volume fraction will have a significant difference in the maximum axial stress of the fiber phase, interface phase, and shear stress at the upper and lower boundaries of the interface phase.

When axial stress and shear stress are measured and their magnitudes and variations are compared for SMA fibers and graphite at room temperature, it is clear that the changes in volume fraction of the two materials have similar stress distribution properties in the fiber phase and interface phase. Therefore, setting the weight of the graphite in the material appropriately can lower the cost of SMA. The minimum axial stress in the interface phase is mainly determined by the pre-strain and volume fraction of SMA fibers, while the influence of graphite volume fraction on the minimum axial stress in the interface phase is relatively small.

## 4. Experimental verification

### 4.1 Preparation of experimental specimens

The graphite/epoxy resin composite specimens embedded with SMAs will be manufactured using the pouring technique. The matrix material is YDF-170 epoxy resin and 325 mesh graphite powder. Adding graphite powder to the epoxy resin matrix is beneficial for improving the strength and thermal conductivity of the matrix material. The curing agent is 2-ethyl-4-methylimidazole, with a mass ratio of 6%. Two specifications of SMA (nickel-titanium alloy wire), with diameters of 0.5 mm and 0.7 mm, are available for selection. Sandpaper was selected to sand the surface of SMA fibers to increase the bonding strength of the fiber and matrix interface and for ultrasonic cleaning of residual stains on the fiber surface.

The mold design of this experiment is based on the conditions of substrate pouring and the high-temperature curing process. According to the test size standards of the tensile test, the total length of the mold is 205 mm, and the gauge length of the uniaxial specimen is 75mm, with a diameter of 12mm and a depth of 3 mm. Create a slot for the fitting of nickel-titanium alloy wires at the end with dimensions of 15 mm × 12 mm × 3 mm. The cutting length of nickel-titanium-alloy wire is 200 mm. According to the mold specifications, three sets of matrix material specimens with different graphite volume fractions were prepared: (1) pure epoxy resin matrix specimens; (2) 2% graphite + epoxy resin composite specimen; (3) 5% graphite + epoxy resin composite specimen. Four sets of SMA composite material specimens with different volume contents of SMA fibers were prepared: (1) three SMA fiber-reinforced composite specimens with a diameter of 0.5 mm; (2) five SMA fiber-reinforced composite specimens with a diameter of 0.5 mm; (3) three SMA fiber-reinforced composite specimens with a diameter of 0.7 mm; and (4) five SMA fiber-reinforced composite specimens with a diameter of 0.7 mm. The size of the test piece is 175 mm × 12 mm × 3 mm. To help with end restraint, ceramic clay is buried in the grooves at both ends of the specimen to fix the end of the nickel-titanium alloy wire.

**Fig 9:**
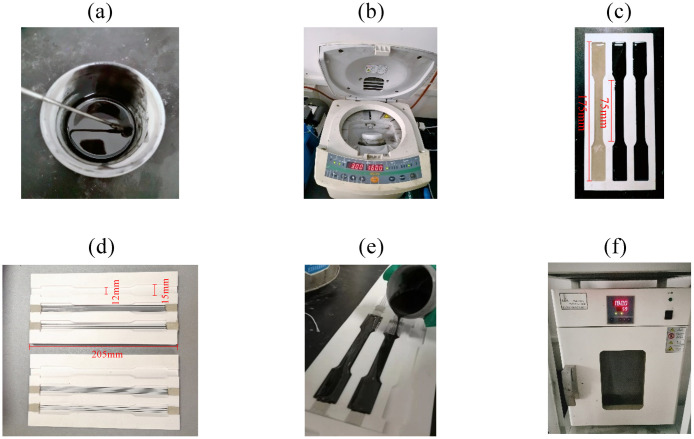
Preparation process flow of matrix and SMAC specimens: (a) Matrix liquid mixing and preparation; (b) Mixing, dispersion and defoaming; (c) Matrix mold pouring; (d) Mold making of SMAC specimens; (e) SMAC mold pouring; (f) Drying oven curing molding.

Fig 9 shows the process flow for preparing SMAC specimens. Mix composite matrix materials with a graphite volume fraction of 2% and 5% corresponding to a graphite mass fraction of 3.01% and 7.41%, respectively. Introduce graphite powder and curing agent into a container containing epoxy resin and manually stir evenly for 20 minutes. Heating during the stirring process can accelerate fusion. Subsequently, the mixture is subjected to stirring, dispersion, and defoaming treatment using an ARE-310 rotary mixer, which ensures the even distribution of the graphite and curing agent in the substrate within the high-viscosity epoxy resin while simultaneously removing bubbles. To complete the preparation of substrate specimens, pour the prepared epoxy resin solution, 2% graphite + epoxy resin solution, and 5% graphite + epoxy resin solution into the mold. To prepare SMAC specimens, the nickel-titanium alloy wire must be securely fixed in the designated mold, and the end should be tightly compacted with clay during the sealing process to ensure the stability of the wire during pouring. The pouring of the substrate solution starts from one end along the direction of the alloy wire and is unidirectional and uniform, avoiding bubbles or gaps near the wire during the pouring process. After pouring, the sample is placed in an electric blast drying oven for curing. The curing process is divided into two stages: the poured sample is dried and cured for 2 hours at a temperature of 60°*C*, and then dried and cured for 8 hours at a temperature of 150°*C*. After the solidification has finished and cooled, it is heated to 120°*C*, at which point the prepared specimen is demolded and removed.

**Fig 10:**
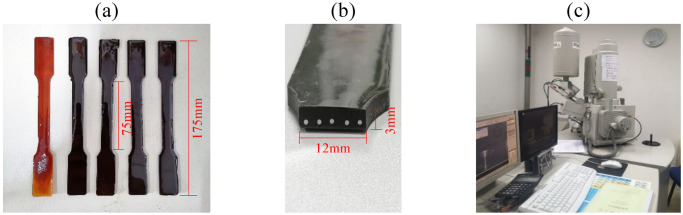
SMAC cross-section samples and testing instruments: (a) Demolding specimen; (b) SMAC laser cutting section; (c) Field emission scanning electron microscope.

Before testing the elastic modulus of the specimen, evaluate the bonding state between the SMA fiber and matrix interface. Laser cut the SMAC specimen along the gauge section and polish the section. A field emission scanning electron microscope was used to examine the surface morphology of the cross-section of SMA fiber-reinforced composite specimens that had been laser cut. The test specimens and instruments are shown in Fig 10.

**Fig 11:**
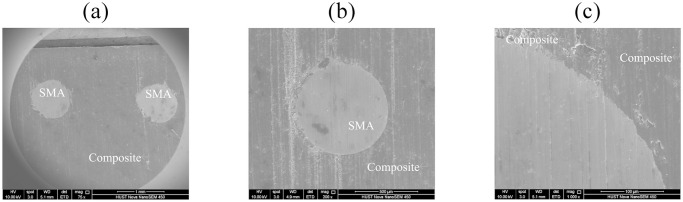
Morphology of Connection between SMA Composite Fiber and Substrate: (a) Micromorphology of two SMA fibers; (b) Microscopic morphology of one SMA fiber; (c) Enlarged view of connection interface morphology.

Fig 11 shows the bonding morphology of the fiber-matrix interface obtained by field emission scanning electron microscopy. The circular area is SMA fibers, while the other areas are composite substrates of graphite and epoxy resin. It can be seen from the figure that the SMA fibers are well connected to the substrate.

### 4.2 Macroscopic elastic modulus measurement

**Fig 12:**
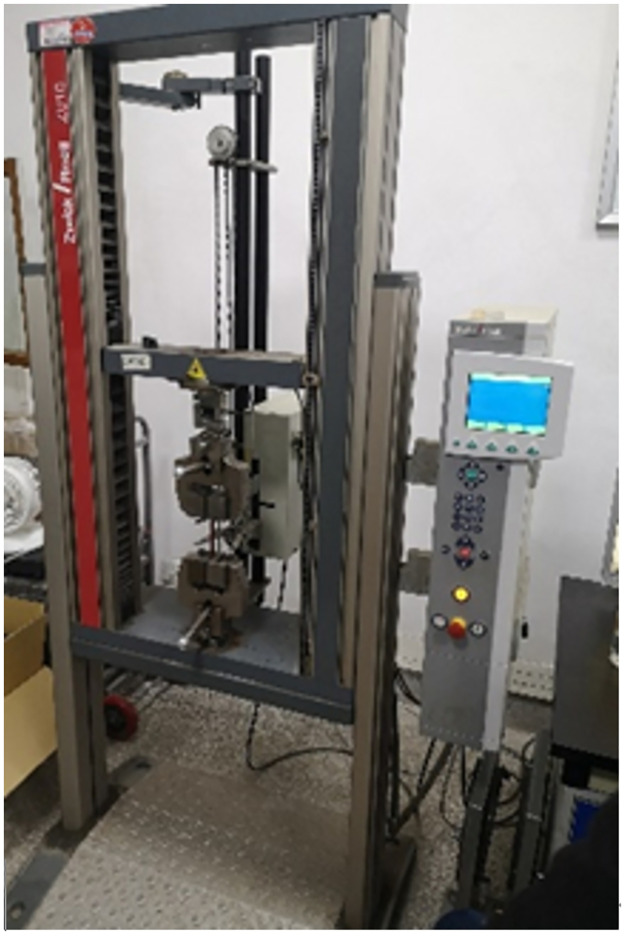
Electronic universal testing machine.

**Fig 13:**
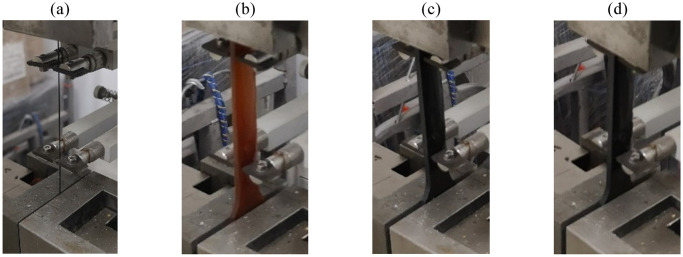
Tensile Experiment Instrument and Test Diagram of Tensile Specimens: (a) SMA wire with a diameter of 0.7 mm; (b) Epoxy resin; (c) 2% graphite + epoxy resin; (d) Three SMA wires with a diameter of 0.7 mm + 2% graphite + epoxy resin.

There are eight various types of specimens used in this study’s samples for elastic modulus testing, including SMA fiber filaments, epoxy resin matrix, graphite + epoxy resin matrix, and SMA + graphite + epoxy resin. Three samples were made for each type of specimen and subjected to tensile testing on the electronic universal testing machine (CMT4104 model) using the ISO 527 tensile strength standard, as shown in Figs 12 and 13. The experimental test results of elastic modulus are the average values of three samples, as shown in the second column of Table 2.

**Table 2:** Comparison of experimental and theoretical results of macroscopic elastic modulus.

Sample category	Elastic modulus (GPa)			
	Specimen test mean	Theoretical	Error (%)	Standard deviation
SMA fiber with a diameter of 0.7 mm	47.70	/	/	/
Epoxy resin matrix	2.89	/	/	/
2% graphite + epoxy resin	3.08	3.03	1.62	0.06
5% graphite + epoxy resin	3.44	3.25	5.52	0.17
Three pieces of 0.5 mm diameter SMA+2% graphite + epoxy resin	4.15	4.11	0.96	0.05
Five pieces of 0.5 mm diameter SMA+2% graphite + epoxy resin	4.59	4.54	1.09	0.06
Three pieces of 0.7 mm diameter SMA+2% graphite + epoxy resin	4.66	4.68	0.43	0.02
Five pieces of 0.7 mm diameter SMA+2% graphite + epoxy resin	5.13	5.17	0.78	0.04

At the same time, the macroscopic elastic modulus of graphite + epoxy resin composite material and SMA + graphite + epoxy resin composite material samples were numerically calculated according to the theoretical model established in the second section, and the results are shown in the third column of Table 2.

The theoretical values are in good agreement with the measured values, as shown by the comparison in Table 2 between the Specimen test mean and the theoretically predicted calculation findings. The results listed above demonstrate the viability of the constitutive model for SMA-reinforced composite materials developed in this article.

## 5. Conclusions

For the constitutive model of non-uniform SMA composites with tensile pre-strain, a three-phase coaxial cylindrical equivalent model that takes into account the interfacial phase and SMA tensile pre-strain is built in this paper. The axial stress and interphase shear stress equations of the SMA composite model are derived. The macroscopic effective modulus of the SMA composite and the stress characteristics of the fiber and interfacial phases of the SMA are determined. The conclusions are as follows:

1. The axial stress of the interface phase of three-phase cylindrical cells was found through numerical simulation to be less than the axial stress of SMA fibers and greater than the axial stress of the matrix. The temperature and pre-strain rise with increasing volume fraction, and most of the shear stress is concentrated at the end.
2. In terms of pre strain, high temperature has the greatest impact on the axial stress of SMA fiber phase and the related forces at the contact point. The largest axial and interfacial stresses are seen in SMA fibers at 10% SMA volume fraction and 5% pre-strain.
3. With a maximum error of less than 1.1%, the theoretical predictions of the macroscopic elastic modulus and elastic modulus of the SMA/graphite epoxy composite material introduced by the interface phase were found to be in good agreement with experimental test values after the SMA/graphite epoxy composite material was prepared.

Additionally, while the fact that this research has produced a more thorough technical approach for determining the equivalent macroscopic effective modulus of SMA composite materials and the analysis of the stress characteristics of SMA fiber phases and interface, there are still limitations and opportunities need to be addressed in further studies: (1) Considering that SMA is a smart material controlled by temperature and magnetic fields, investigating how external influences affect the mechanical properties of SMA composites offers prospects for research and applications; (2) SMA can be embedded in a variety of matrix materials. This work includes short fiber distribution, hybrid distribution, SMA polymer types, and long fiber penetration embedding. More research is still required to completely comprehend their precise constitutive models and analytical techniques.

## Supporting information

S1 Appendix (DOCX)

S1 Data The primary data. (XLSX)
