# Supplementary material for: Interface stress transfer model and modulus parameter equivalence method for composite materials embedded with tensile pre-strain shape memory alloy fibers
Source: PLoS One. 2024 May 14;19(5):e0302729. doi: 10.1371/journal.pone.0302729 (PMC11093366; doi:10.1371/journal.pone.0302729)
Supplement: S1 Appendix — (DOCX) [file pone.0302729.s001.docx]

**Appendix A**

The relevant parameter models in the stress expression of SMA composite materials include $A_{p}(p=1,2,\ldots,6)$，$B_{q}(q=1,2,\ldots,6)$ and $C_{t}(t=1,2,3,4)$.

| $A_{p}=\left\{ \begin{aligned} \begin{matrix} \frac{r_{i}^{2}}{r_{i}^{2}-r_{SMA}^{2}}ln\left( \frac{r_{i}}{r_{SMA}} \right)-\frac{1}{2} & ,p=1 \end{matrix} \\ \begin{matrix} \frac{r_{SMA}^{2}}{r_{i}^{2}-r_{SMA}^{2}}ln\left( \frac{r_{i}}{r_{SMA}} \right)-\frac{1}{2} & ,p=2 \end{matrix} \\ \begin{matrix} \frac{r_{m}^{2}}{r_{m}^{2}-r_{i}^{2}}ln\left( \frac{r_{m}}{r_{i}} \right)-\frac{1}{2} & ,p=3 \end{matrix} \\ \begin{matrix} \frac{r_{m}^{4}ln\left( r_{m}/r_{i} \right)-\left( 3r_{m}^{2}-r_{i}^{2} \right)\left( r_{m}^{2}-r_{i}^{2} \right)/4}{\left( r_{m}^{2}-r_{i}^{2} \right)^{2}} & ,p=4 \end{matrix} \\ \begin{matrix} \frac{r_{i}^{4}ln\left( r_{i}/r_{SMA} \right)-\left( 3r_{i}^{2}-r_{SMA}^{2} \right)\left( r_{i}^{2}-r_{SMA}^{2} \right)/4}{\left( r_{i}^{2}-r_{SMA}^{2} \right)^{2}} & ,p=5 \end{matrix} \\ \begin{matrix} \frac{r_{i}^{2}r_{SMA}^{2}ln\left( r_{i}/r_{SMA} \right)-\left( r_{i}^{2}-r_{SMA}^{2} \right)/4}{\left( r_{i}^{2}-r_{SMA}^{2} \right)^{2}} & ,p=6 \end{matrix} \end{aligned} \right.$ | (A-1) |
| --- | --- |
| $B_{q}=\left\{ \begin{aligned} \begin{matrix} \frac{A_{4}\left( A_{5}-A_{1} \right)}{B} & q=1 \end{matrix} \\ \begin{matrix} \frac{A_{1}A_{4}}{B} & q=2 \end{matrix} \\ \begin{matrix} \frac{\left( A_{1}A_{6}-A_{2}A_{5} \right)}{B}\frac{G_{m}}{G_{i}} & q=3 \end{matrix} \\ \begin{matrix} \frac{\left( A_{5}-A_{1} \right)\left( A_{4}-A_{3} \right)}{B} & q=4 \end{matrix} \\ \begin{matrix} \frac{A_{1}\left( A_{4}-A_{3} \right)}{B} & q=5 \end{matrix} \\ \begin{matrix} \frac{A_{3}}{A_{4}}+\frac{\left( A_{4}-A_{3} \right)\left( A_{1}A_{6}-A_{2}A_{5} \right)}{A_{4}B}\frac{G_{m}}{G_{i}} & q=6 \end{matrix} \end{aligned} \right.$ | (A-2) |
| $B=A_{4}A_{5}-\left( A_{2}A_{5}-A_{1}A_{6} \right)\frac{G_{m}}{G_{i}}$ | (A-3) |
| $C_{t}=\left\{ \begin{aligned} \begin{matrix} \frac{\text{1}}{\text{r}_{\text{SMA}}^{\text{2}}}\frac{\text{1}}{\text{A}_{\text{1}}} & t=1 \end{matrix} \\ \begin{matrix} \frac{\text{r}_{\text{m}}^{\text{2}}}{\text{r}_{\text{m}}^{\text{2}}\text{-}\text{r}_{\text{i}}^{\text{2}}} & t=2 \end{matrix} \\ \begin{matrix} \frac{\text{1}}{\text{r}_{\text{i}}^{\text{2}}\text{-}\text{r}_{\text{SMA}}^{\text{2}}} & t=3 \\ \frac{\text{r}_{\text{SMA}}^{\text{2}}}{\text{r}_{\text{m}}^{\text{2}}\text{-}\text{r}_{\text{i}}^{\text{2}}} & t=4 \end{matrix} \end{aligned} \right.$ | (A-4) |

The coefficients $K_{1}$ , $K_{2}$ , $H_{1}$ , $H_{2}$ , $T_{1}$ , $T_{2}$ , $\text{J}_{\text{1}}$and $J_{2}$ in equations (28) to (32) are expressed as follows

| $K_{1}=\frac{\left( M_{13}-{\frac{\text{r}_{\text{i}}^{\text{2}}\text{-}r_{SMA}^{2}}{\text{r}_{\text{m}}^{\text{2}}}M}^{'} \right)\left( M^{''}-M_{21}\frac{\text{ε}_{SMA}^{pre}\text{-}\text{ε}_{\text{L}}\text{ξ}_{\text{s}}}{\text{σ}_{\text{c}}} \right)-\left( M_{23}-{\frac{\text{r}_{\text{i}}^{\text{2}}\text{-}r_{SMA}^{2}}{\text{r}_{\text{m}}^{\text{2}}}M}^{''} \right)\left( M^{'}{-M}_{11}\frac{\text{ε}_{SMA}^{pre}\text{-}\text{ε}_{\text{L}}\text{ξ}_{\text{s}}}{\text{σ}_{\text{c}}} \right)}{\left( M_{12}-{\frac{r_{SMA}^{2}}{\text{r}_{\text{m}}^{\text{2}}}M}^{'} \right)\left( M_{23}-{\frac{\text{r}_{\text{i}}^{\text{2}}\text{-}r_{SMA}^{2}}{\text{r}_{\text{m}}^{\text{2}}}M}^{''} \right)-\left( M_{13}-{\frac{\text{r}_{\text{i}}^{\text{2}}\text{-}r_{SMA}^{2}}{\text{r}_{\text{m}}^{\text{2}}}M}^{'} \right)\left( M_{22}-{\frac{r_{SMA}^{2}}{\text{r}_{\text{m}}^{\text{2}}}M}^{''} \right)}$ | (A-5) |
| --- | --- |
| $K_{2}=\frac{\left( M_{22}-{\frac{r_{SMA}^{2}}{\text{r}_{\text{m}}^{\text{2}}}M}^{''} \right)\left( M^{'}{-M}_{11}\frac{\text{ε}_{SMA}^{pre}\text{-}\text{ε}_{\text{L}}\text{ξ}_{\text{s}}}{\text{σ}_{\text{c}}} \right)-\left( M_{12}-{\frac{r_{SMA}^{2}}{\text{r}_{\text{m}}^{\text{2}}}M}^{'} \right)\left( M^{''}-M_{21}\frac{\text{ε}_{SMA}^{pre}\text{-}\text{ε}_{\text{L}}\text{ξ}_{\text{s}}}{\text{σ}_{\text{c}}} \right)}{\left( M_{12}-{\frac{r_{SMA}^{2}}{\text{r}_{\text{m}}^{\text{2}}}M}^{'} \right)\left( M_{23}-{\frac{\text{r}_{\text{i}}^{\text{2}}\text{-}r_{SMA}^{2}}{\text{r}_{\text{m}}^{\text{2}}}M}^{''} \right)-\left( M_{13}-{\frac{\text{r}_{\text{i}}^{\text{2}}\text{-}r_{SMA}^{2}}{\text{r}_{\text{m}}^{\text{2}}}M}^{'} \right)\left( M_{22}-{\frac{r_{SMA}^{2}}{\text{r}_{\text{m}}^{\text{2}}}M}^{''} \right)}$ | (A-6) |
| $H_{1}=\frac{R_{1}\left( 1-K_{1} \right)-R_{1}R_{2}\left( 1-K_{2} \right)}{R_{1}-R_{2}}$ | (A-7) |
| $H_{2}=\frac{R_{1}R_{2}\left( 1-K_{2} \right)-R_{2}\left( 1-K_{1} \right)}{R_{1}-R_{2}}$ | (A-8) |
| $R_{1}=\left( M_{12}-M_{23}+{\frac{\text{r}_{\text{i}}^{\text{2}}\text{-}r_{SMA}^{2}}{\text{r}_{\text{m}}^{\text{2}}}M}^{''}-{\frac{r_{SMA}^{2}}{\text{r}_{\text{m}}^{\text{2}}}M}^{'}-P \right)/2\left( M_{22}-{\frac{r_{SMA}^{2}}{\text{r}_{\text{m}}^{\text{2}}}M}^{''} \right)$ | (A-9) |
| $R_{2}=\left( M_{12}-M_{23}+{\frac{\text{r}_{\text{i}}^{\text{2}}\text{-}r_{SMA}^{2}}{\text{r}_{\text{m}}^{\text{2}}}M}^{''}-{\frac{r_{SMA}^{2}}{\text{r}_{\text{m}}^{\text{2}}}M}^{'}+P \right)/2\left( M_{22}-{\frac{r_{SMA}^{2}}{\text{r}_{\text{m}}^{\text{2}}}M}^{''} \right)$ | (A-10) |
| $T_{1}=\sqrt{\left( M_{12}+M_{23}-{\frac{r_{SMA}^{2}}{\text{r}_{\text{m}}^{\text{2}}}M}^{'}-{\frac{\text{r}_{\text{i}}^{\text{2}}\text{-}r_{SMA}^{2}}{\text{r}_{\text{m}}^{\text{2}}}M}^{''}-P \right)/2}$ | (A-11) |
| $T_{2}=\sqrt{\left( M_{12}+M_{23}-{\frac{r_{SMA}^{2}}{\text{r}_{\text{m}}^{\text{2}}}M}^{'}-{\frac{\text{r}_{\text{i}}^{\text{2}}\text{-}r_{SMA}^{2}}{\text{r}_{\text{m}}^{\text{2}}}M}^{''}+P \right)/2}$ | (A-12) |
| $P=\left[ \begin{aligned} \left( M_{12}-{\frac{r_{SMA}^{2}}{\text{r}_{\text{m}}^{\text{2}}}M}^{'} \right)^{2}+\left( M_{23}-{\frac{\text{r}_{\text{i}}^{\text{2}}\text{-}r_{SMA}^{2}}{\text{r}_{\text{m}}^{\text{2}}}M}^{''} \right)^{2}+4\left( M_{13}-{\frac{\text{r}_{\text{i}}^{\text{2}}\text{-}r_{SMA}^{2}}{\text{r}_{\text{m}}^{\text{2}}}M}^{'} \right)\left( M_{22}-{\frac{r_{SMA}^{2}}{\text{r}_{\text{m}}^{\text{2}}}M}^{''} \right) \end{aligned}-2\left( M_{12}-{\frac{r_{SMA}^{2}}{\text{r}_{\text{m}}^{\text{2}}}M}^{'} \right)\left( M_{23}-{\frac{\text{r}_{\text{i}}^{\text{2}}\text{-}r_{SMA}^{2}}{\text{r}_{\text{m}}^{\text{2}}}M}^{''} \right) \right]^{\frac{1}{2}}$ | (A-13) |
| $J_{1}=\frac{\left( 1-K_{1} \right)-R_{2}\left( 1-K_{2} \right)}{R_{1}-R_{2}}$ | (A-14) |
| $J_{2}=\frac{R_{1}\left( 1-K_{2} \right)-\left( 1-K_{1} \right)}{R_{1}-R_{2}}$ | (A-15) |
